# Supplementary material for: High-throughput robust single-cell DNA methylation profiling with sciMETv2
Source: Nat Commun. 2022 Dec 9;13:7627. doi: 10.1038/s41467-022-35374-3 (PMC9734657; doi:10.1038/s41467-022-35374-3)
Supplement: Supplementary file 1 — Supplementary Information [file 41467_2022_35374_MOESM1_ESM.pdf]

a sciMETv2 Processing Flowchart

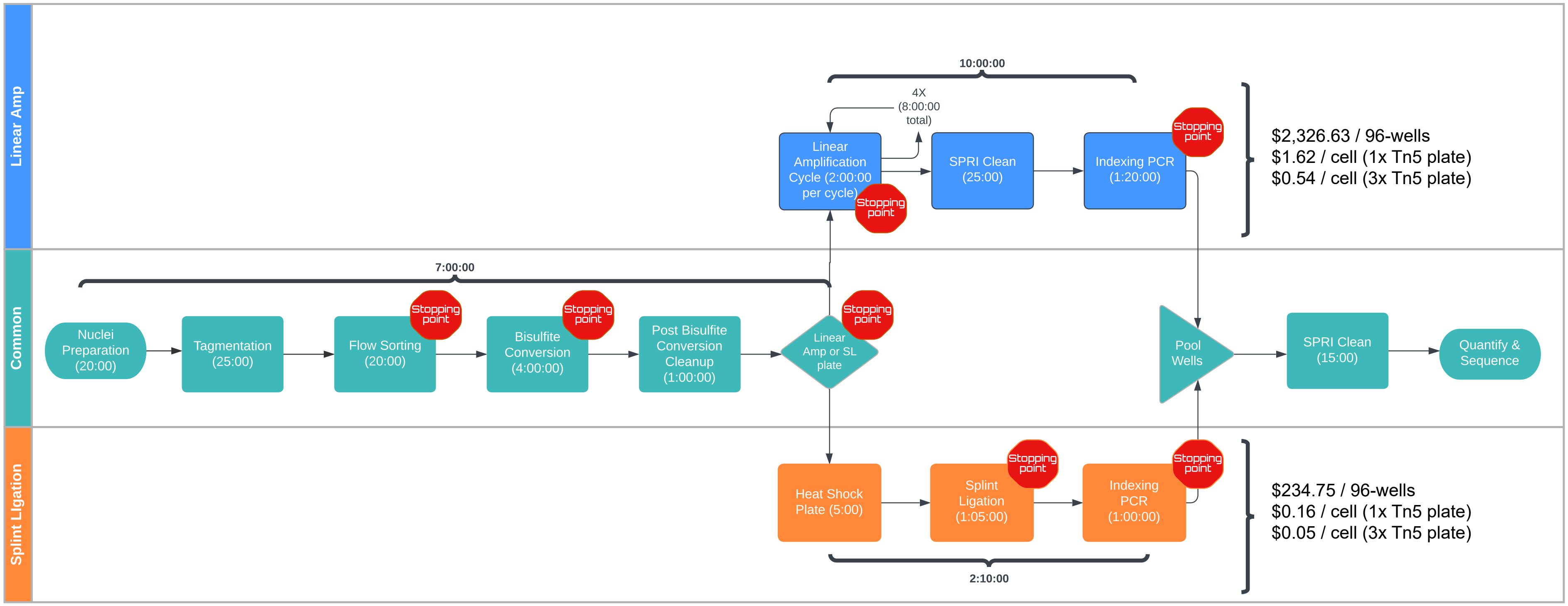

b sciMETv2 Cost Breakdown

Linear Amplification

Reagent	Cost/well	Cost/plate*
10X NEB Buffer 2.1	$0.03	$2.86
dNTP Mix	$0.17	$18.15
100 µM 9H Random Prime	$0.10	$2.14
TwinTec PCR Plate	$0.63	$10.00
Klenow Exo -	$4.42	$486.20
2nd-4th linear amp cycles:		
100uM 9H Primer	$0.15	$16.02
10mM dNTPs	$0.02	$1.72
Klenow exo-	$13.26	$1,458.60
10X NEB buffer 2.1	$0.01	$0.86
Omega Bio-tek SPRI bead	$0.50	$54.85
TwinTec PCR Plate	$0.10	$10.00
Q5U 2X MM	$1.98	$217.80
EVAgreen 100X	$0.10	$10.89
10uM TS i5 Primer	$0.02	$2.69
10uM TS i7 Primer	$0.02	$2.69
Tips (estimated)	$0.32	$31.16
**Total**	**$21.81**	**$2,326.63**

Splint Ligation

Reagent	Cost/well	Cost/plate*
20ng ET SSB	$0.07	$7.44
TwinTec PCR plate	$0.10	$10.00
PEG8000, 50%	$0.00	$0.30
1,3 PrD	$0.01	$0.59
SCR Buffer	$0.00	$0.00
1M DTT	$0.01	$0.94
100mM ATP	$0.00	$0.32
T4 PNK	$0.11	$12.54
T4 Ligase	$0.65	$71.50
0.75uM SL Adapter	$0.02	$2.06
VeraSeq PCR Mix	$0.84	$91.92
EVAGreen 100x	$0.10	$10.89
10uM TS i5 Primer	$0.01	$1.35
10uM TS i7 Primer	$0.01	$1.35
Tips (estimated)	$0.24	$23.56
	**$2.17**	**$234.75**

Nominal Cost/cell

	Linear Amplification	Splint Ligation
**Using 1x Tn5 plate**	**$1.62**	**$0.16**
**Using 3x Tn5 plates**	**$0.54**	**$0.05**

*Cost/plate is for 110 wells in order to include excess reagents used for making a master mix.

Reagent Costs

Reagent	Cost	[Reagent]	Cost/Unit
NEB ET SSB	$169.00	500 ng/ul	$0.00
NEB buffer 2.1	$26.00	10X	$0.01 $/ng
NEB dNTPs	$66.00	10mM	$0.08 $/ul
100 µM 9H Random Primer	$252.45	100uM	$0.49 $/ul
Eppendorf TwinTec PCR pla	$100.00	10/ea	$10.00 $/ul
Splint	$207.75	100uM	$1.78 $/ea
Splint Adapter	$92.55	100uM	$0.72 $/100pmol
Enzymatics Klenow Exo -*	$442.00	5U/ul	$2.21 $/100pmol
Omega Bio-tek SPRI beads	$3,835.80	500mL	$0.01 $/ul
NEB Q5U 2x MM	$495.00	250	$1.98 $/ul
EVAGREEN 100X	$198.00	2000X	$0.20 $/rxn
10uM TS i7 Primers (12)	$146.83	100uM	$0.01 $/ul 100X
10uM TS i5 primers (8)	$97.89	100uM	$0.01 $/ul 10uM
50% PEG 8000	$166.00	50%, 100mL	$0.00 $/ul 10uM
SCR Buffer	$1.29	100mL	$0.00 $/ul
DTT 1M (Thermo, P2325)	$85.50	1M, 1mL	$0.09 $/ul
ATP 100mM	$147.00	100mM, 5mL	$0.03 $/ul
T4 PNK	$228.00	10U/ul	$0.91 $/ul
T4 Ligase	$260.00	2kU/ul	$5.20 $/ul
1,3 Propanedio (Merck)	$27.80	100%	$0.01 $/ul
Tips (estimated cost)			$0.04 $/tip
VeraSeqUltra	$1,671.20	2000 U	$0.84 $/rxn

* In sciMETv1 Klenow was available at a substantially reduced price (approximately 1/8th the current price). That reagent is no longer available, and the Enzymatics klenow is used instead, increasing costs substantially for the LA method.

Supplementary Figure 1: Protocol Flowchart and Cost Breakdown | a. Flowchart of the sciMETv2 workflows including time estimates and safe stopping points with a comparison of per-plate costs for the linear amplificaiton (LA) and splint ligation (SL) workflows. **b.** Detailed cost breakdown for each method by reagent.

Supplementary Note 1: sci-METv2 Protocol

Ruth V. Nichols O'Connell – 220802

Nuclei Preparation, Tagmentation & Sorting

1. Prepare 4X Taps-TD (see appendix for the recipe) fresh on the day of the experiment.
2. Incubate the M-Digestion Buffer at 37°C until the precipitate dissolves.
3. Prepare the post sort plates with the following in each well (total volume is 2 µL):

	1X	110X	220X	440X
**M-Digestion Buffer**	1	110	220	440
**Qiagen Proteinase K**	0.07	7.7	15.4	30.8
**dH_2O**	0.93	102.3	204.6	409.2

4. Isolate nuclei in NIB-H (see appendix for the recipe) with protease inhibitors added.
5. Quantify nuclei
6. Start heating the 10% SDS at 37°C or higher to dissolve any precipitates.
7. Split nuclei into 1 million nuclei aliquots.
8. Combine each aliquot with 47 uL 16% formaldehyde and enough NIB-H for a total volume of 1 mL. Incubate for 10 minutes at Room Temperature with occasional mixing.
9. Add 47 uL 2.5M glycine. Mix by inversion and incubate for 5 minutes on ice.
10. Spin down at 500xG at 4°C for 5 minutes and resuspend in 970 uL NDM (ScaleBiosciences CAT# 230031) to disrupt nuclei. Incubate for 20 minutes at 37°C.
11. Spin down and resuspend in NIB-H. Recombine aliquots. Quantify nuclei. Nuclei can be left overnight at 4°C.
12. Tagment 1 plate of nuclei with the following (total volume for each well is 10 µL):

	1X	110X
**5000 nuclei**		
**4X-Taps TD**	2.5	275
**mC-Tn5 (5 uM) – Scale Bio.**	2	---
**NIB-H (+protease inhibitors)**	Enough to fill to 10 µL	

13. Incubate at 55C for 15 minutes. Put on ice.
14. Pool nuclei, run through a cell strainer and add 3 uL 5 mg/mL DAPI
15. Sort 15 nuclei per well into the post-sort plates. Spin down the plate.
16. Incubate at 50C for 20 minutes to digest nuclei. Spin down the plate. Plates can be frozen at this stage.

Bisulfite Conversion

1. Add the following to 1 bottle of CT Conversion Reagent:
   7.9 mL M-Solubilization Buffer
   3.0 mL M-Dilution Buffer
2. Shake vigorously to dissolve by taping upright onto lab shaker and setting to max speed.
3. Once dissolved, add 1.6 mL M-Reaction Buffer to the bottle.
4. If your plate of nuclei was frozen, spin it down prior to opening.
5. To each well of your plate of digested nuclei, add 15 µL of the prepared CT Conversion Reagent. Use new tips every time. Pipette up and down 8 times to mix. Spin down the plate.
6. Incubate with the following program:
   98°C 8 minutes
   64°C 3.5 hours (210 minutes)
   4°C hold
7. Spin down the plate. Plate can be frozen at this stage.

Post-Bisulfite Conversion Cleanup

1. Spin down the plate.
2. Add 80 µL M-Binding Buffer to each well of your plate. Pipette to mix and transfer to a 96-well Zymo-Spin I-96 Plate (Shallow well)
3. Spin down at max speed (~2200 xG) for 8 minutes using a 'crappy' plate underneath to catch the flow-through. Discard flow through by dumping and blot plate to dry.
4. Add 100 µL M-Wash Buffer (or 80% EtOH) to each well. Spin at the above speed and time. May need to increase the time and spin twice to be sure to remove the Wash Buffer. Discard the Wash Buffer as above.
5. Add 50 µL M-Desulphonation Buffer to each well. Incubate for 15 minutes at room temperature. Spin at the same speed and time to remove the buffer.
6. Add 200 µL M-Wash Buffer (or 80% EtOH) to each well. Spin out the Wash Buffer as in above. May need to increase the time and spin twice to be sure to remove the Wash Buffer.
7. Spin onto an empty plate to completely dry the columns.
8. Preheat Buffer EB at 55°C and have a thermo-mixer preheated to 55°C
9. Assemble either a Linear Amp (LA) plate or a Splint Ligation (SL) plate as follows:
   a. **LA plate**

	**1X**	**110X**
**dH$_2$O**	17.8	1760
**10X NEB Buffer 2.1**	5	550
**10 mM dNTPs**	2	220
**100 µM 9H Random Primer (scMET_5SpC3_H9_IPE1)**	0.2	22

   b. **SL plate**
   1 uL 20 ng/µL ET-SSB in each well
   Note: Stock ET-SSB is diluted in SSB Dilution Buffer (see appendix)

10. Put the Zymo-Spin column plate onto either an LA or SL plate
    a. **If LA**, add 25 µL preheated Buffer EB to each column in the plate. Put the assembled plates together onto preheated thermo-mixer and incubate for 4 minutes at 55°C
    b. **If SL**, add 5 µL preheated Buffer EB to each column in the plate. Put the assembled plates together onto preheated thermo-mixer and incubate for 4 minutes at 55°C
11. Spin at above speed and time to elute. Plate can be frozen at this stage.

****If doing SL, skip the Linear Amplification sections****

<u>Linear Amplification – 1st cycle</u>

1. Spin down the plate.
2. Have an ice bucket ready at the thermocycler.
3. Heat shock the plate at 95°C for 45 seconds then place on ice until cool (approximately 2 minutes). Quick-Spin down the plate at 4°C.
4. Add 10U Klenow exo$^-$ (2 uL for Enzymatics brand) to each well. Seal plate
5. Put plate back onto thermocycler with the following program:
   4°C 5 minutes
   Ramp 1°C every 15 seconds until 37°C is reached
   37°C 90 minutes
   4°C hold
6. Spin down the plate. Plate can be frozen at this point.

<u>Linear Amplification –2nd – 4th cycles</u>

1. Spin down the plate if it was frozen.
2. Heat shock the plate at 95°C for 45 seconds then place on ice until cool (approximately 2 minutes). Quick-Spin down the plate at 4°C.
3. Add the following to each well while on ice:

	**1X**	**110X**
**100 µM 9H Random Primer (scMET_5SpC3_H9_IPE1)**	0.1	11
**10 mM dNTPs**	1	110
**10X NEB Buffer 2.1**	0.5	55
**dH_2O**	1.65	181.5
**Klenow exo$^-$ 5U/µL (Enzymatics)**	2	220

4. Put plate back onto thermocycler with the following program:
   4°C 5 minutes
   Ramp 1°C every 15 seconds until 37°C is reached
   37°C 90 minutes
   4°C hold
5. Spin down the plate. Plate can be frozen at this point.
6. Repeat Step 1 – 5 twice more for a total of 3 additional LA cycles (4 total).

Linear Amplification – Indexing PCR

1. Spin down the plate.
2. Perform a plate clean-up of 1.1:1 SPRI clean (depending on evaporation, this is ~65 µL SPRI per well). Use new tips for each well.
3. Elute with 21 µL Buffer EB into a plate containing the following (final volume is 50 µL):

	1X	110X
**Q5U 2X Master Mix**	25	2750
**10 µM TruSeq i5 primers**	2	---
**10 µM TruSeq i7 primers**	2	---
**EvaGreen 100X**	0.5	55

4. Pipette or vortex to mix. Spin down.
5. Use the following cycling protocol on the CFX (cycling the middle 3 steps):
   95°C 2 minutes
   94°C 80 seconds
   65°C 30 seconds
   72°C 30 seconds
   72C 10 seconds *plate read
6. Good preps have gotten 13-15 cycles
7. Pool 10 µL per well. Perform a column clean-up and then a 1:1 SPRI cleanup.
8. Do a Qubit, TapeStation, Sequence etc.

****If doing LA, skip the Splint Ligation sections****

Splint Ligation - SL

1. Spin down the plate
2. Take out all SL reagents, putting everything except the enzymes at room temperature.
3. Incubate the 50% PEG 8000 at 50C. Once heated take the required aliquot and place into a new master mix tube. Allow the tube to return to room temperature.
4. Assemble the SL Master Mix by adding reagents to the room temperature PEG tube. Add the enzymes last, just before pipetting to the receiving plate. **DO NOT place master mix on ice. The PEG will become very difficult to pipette**

Original Recipe	**1X**	**110X**
**50% PEG 8000**	4	440
**SCR Buffer**	0.75	82.5
**DTT 1M**	0.1	11
**ATP 100 mM**	0.1	11
**T4 PNK (10,000 U/mL, NEB)**	0.125	13.75
**T4 Ligase (2,000,000 U/mL, NEB)**	0.125	13.75

5. Have an ice bucket ready at the thermocycler
6. Heat shock the plate at 95°C for 3 minutes. Place directly on ice for 2 minutes.
7. Quick-Spin down the plate at 4°C. Place on ice.
8. If not already done, add the PNK and Ligase to the SL Master Mix. DO NOT put on ice. Pulse-Vortex at max speed several times with some spin downs in between.
9. Add 1 µL 0.75 µM pre-annealed P5 adapter to each well of the plate.
10. Remove plate from the ice and put on the bench at Room Temperature.
11. Add 5.2 µL Room Temperature SL Master Mix to each well. **Use P20 tips and pipette slowly**
12. Seal the plate and put on the plate shaker at ~1000 RPM for 5 seconds.
13. Quick-Spin down the plate.
14. Incubate at 37°C for 45 minutes.
15. Incubate at 65°C for 20 minutes to inactivate the ligase.
16. Spin down the plate. Plate can be frozen at this point.

SL – Indexing PCR

1. Spin down the plate.
2. Add the following to each well (final volume is 50 µL). Primers are added individually with a combinatorial index approach:

	1X	110X
**5X VeraSeq GC Buffer**	10	1100
**10 µM dNTPs**	2	220
**VeraSeq ULtra Enzyme**	1.5	165
**dH_2O**	24	2640
**EvaGreen 100X**	0.5	55
**10 µM TruSeq i5 primers**	1	add individually
**10 µM TruSeq i7 primers**	1	add individually

3. Vortex to mix. Spin down.
4. Put the plate on the CFX when sample temperature reaches 98°C. This is to ensure that any possibly active ligase doesn't have a chance to ligate PCR primers.
5. Use the following cycling protocol (cycling the middle 3 steps):
   98°C 30 seconds
   98°C 10 seconds
   57°C 20 seconds
   72°C 30 seconds
   72°C 10 seconds *plate read
6. Good preps have gotten 13-16 cycles.
7. Pool 10 µL per well. Perform a column clean-up and then a 1:1 SPRI cleanup.
8. Do a Qubit, TapeStation, Sequence etc.

Appendix

References & Commercial Reagents

- Ecker protocol (https://www.protocols.io/view/methyl-c-sequencing-of-single-cell-nuclei-snmc-seq-pjvdkn6)[1]
- Mulqueen et al 2018 – sciMET[2]
- Kapp et al 2021 – SL protocol (use supplement)[3]
- Zymo Research Zymo-Spin I-96 Plate (Shallow well) Cat# C2004-SW
- Qiagen Buffer EB Cat# 19086
- Qiagen Proteinase K Cat# 19131
- Zymo Research EZ-DNA Methylation Direct Kit & Reagents:
    - M-Digestion Buffer Cat# D5021-9
    - CT Conversion Reagent Cat# D5003-1
    - M-Solubilization Buffer Cat# D5021-7
    - M-Dilution Buffer Cat# D5002-2
    - M-Reaction Buffer Cat# D5021-8
    - M-Binding Buffer Cat# D5040-3
    - M-Wash Buffer Cat# D5040-4
    - M-Desulphonation Buffer Cat# D5040-5
- Enzymatics Reagents:
    - Klenow (3'-5' exo$^-$) Cat# P7010-LC-L **(for LA only)**
    - VeraSeq ULtra DNA Polymerase Cat# P7520S **(for SL only)**
- NEB Reagents:
    - Q5U Polymerase Cat# M0515L
    - Q5U 2X Master Mix Cat# M0597L **(for LA only)**
    - 10X Buffer 2.1 Cat# B7030 **(for LA only)**
    - **(for SL only):**
        - T4 DNA Ligase (2,000,000 U/mL) Cat# M0202M
        - T4 PNK (10,000 U/mL) Cat# M0201L
        - ET SSB (500 ng/µL) Cat# M2401S
        - T4 RNA Ligase Buffer Cat# B0216L
        - PEG 8000 (50%) Cat# B0216L (from kit)
- ThermoScientific
    - ATP 100mM (cat# R0441)

Oligos

1. Linear amp primer (scMET_5SpC3_H9_IPE1): /5SpC3/TTCCCTACACGACGCTCTTCCGATCT(H1:33340033)(H1)(H1)(H1)(H1)(H1)(H1)(H1)(H1)

2. SL P5_rc adapter (SL_TruSeq_i5_rc): /5Phos/AGATCGGAAGAGCGTCGTGTAGGGAAAGAGTGT/3AmMO/

3. SL splints (currently testing for which is best):
   a. N7 (SL_TruSeq_i5_splint): /5AmMC12/ACACTCTTTCCCTACACGACGCTCTTCCGATCTNNNNNNN/3AmMO/

   b. H7 (SL_TSi5splint_H7): /5AmMC12/ACACTCTTTCCCTACACGACGCTCTTCCGATCT(H:33330034)(H)(H)(H)(H)(H)(H)(H)(H)(H)/3AmMO/

   c. H10 (SL_TSi5splint_H10): /5AmMC12/ACACTCTTTCCCTACACGACGCTCTTCCGATCT(H:33330034)(H)(H)(H)(H)(H)(H)(H)(H)(H)(H)(H)/3AmMO/

Nuclei Preparation & Tagmentation – Buffer Preparation

- **NIB-H** (Store at 4°C): 10 mM HEPES, pH 7.5 [Sigma, Cat. H4034], 10 mM NaCl [Fisher, Cat. M-11624], 3 mM MgCl2 [Sigma, Cat. M8226], 0.1% IGEPAL [v/v; Sigma, I8896], 0.1% Tween-20 [v/v, Sigma, Cat. P7949], and 1× protease inhibitor [Roche, Cat. 11873580001])
- **4X Taps-TD** (Do not store, make fresh): 33mM TAPS pH=8.5 [Sigma, Cat. T5130], 66mM KOAc [Sigma, Cat. P1190], 10mM MgOAc [Sigma, Cat. M5661], 16% DMF [Sigma, Cat. D4551]
- **NDM** ScaleBio Part No. 230031

SL – Buffer Preparation

- **Adapter Dilution Buffer** (Store at -20°C): 1X T4 RNA Ligase Buffer, 0.05% Tween-20
- **SSB Dilution Buffer** (Store at -20°C): 20 mM Tris-HCl (pH 8.0), 20 mM NaCl, 0.5 mM DTT, 0.1 mM EDTA (pH 8.0), 50% Glycerol
- **SCR Buffer** (Store at -20°C): 666 mM Tris-HcL, 132 mM $MgCl_2$

SL – Annealing adapters & splints

1. In PCR tubes add the following:

	1X	
**dH$_2$O**	30.6	
**10X T4 RNA Ligase Buffer (NEB)**	5	
**100 µM P5 adapter (SL_TruSeq_i5_rc)**	6	
**100 µM P5 splint**	8.4	

2. Incubate with the following program:
   95°C 1 minute
   Ramp down to 10°C at 0.1°C per second
   10°C hold
3. This stock is 12 µM. For sciMET2.0 dilute to 0.75 µM in aliquots with 18.75 µL stock + 281.25 µL Adapter Dilution Buffer (1X T4 RNA Ligase Buffer, 0.05% Tween-20). Kapp et al. 2021 recommends limiting the number of freeze-thaw cycles of pre-annealed adapters to 4.

Supplementary References

1. Luo, C. & Ecker, J. Methyl-C sequencing of single cell nuclei: snmC-seq2. https://www.protocols.io/view/methyl-c-sequencing-of-single-cell-nuclei-snmc-seq-8epv58zdv1bz/v1 (2018).
2. Mulqueen, R. M. *et al.* Highly scalable generation of DNA methylation profiles in single cells. *Nat. Biotechnol.* **36**, 428–431 (2018).
3. Kapp, J. D., Green, R. E. & Shapiro, B. A Fast and Efficient Single-stranded Genomic Library Preparation Method Optimized for Ancient DNA. *J. Hered.* **112**, 241–249 (2021).
