# Supplementary material for: High-throughput robust single-cell DNA methylation profiling with sciMETv2
Source: Nat Commun. 2022 Dec 9;13:7627. doi: 10.1038/s41467-022-35374-3 (PMC9734657; doi:10.1038/s41467-022-35374-3)
Supplement: Supplementary file 3 — Description of additional Supplementary File [file 41467_2022_35374_MOESM3_ESM.pdf]

Description of Supporting Information

Supplementary Data 1 | sciMET Oligos. Tables containing the oligonucleotide sequences for the tagmentation and PCR processing steps.

Supplementary Data 2 | sciMET cell information. A tab-delimited gzipped text file containing per-cell statistics for each cell.

Supplementary Data 3 | Cluster-based promoter CG methylation. Methylation levels for gene promoters for each cluster.

Supplementary Data 4 | Neuron cluster-based promoter CG methylation. Methylation levels for gene promoters specifically for neuronal clusters.

Supplementary Data 5 | Neuron cluster-based gene body CH methylation. Methylation levels in the CH context for gene bodies specifically for neuronal clusters.
