# Supplementary material for: High-throughput robust single-cell DNA methylation profiling with sciMETv2
Source: Nat Commun. 2022 Dec 9;13:7627. doi: 10.1038/s41467-022-35374-3 (PMC9734657; doi:10.1038/s41467-022-35374-3)
Supplement: Supplementary file 9 — Reporting Summary [file 41467_2022_35374_MOESM9_ESM.pdf]

nature portfolio

Corresponding author(s): Andrew Adey

Last updated by author(s): Sep 29, 2022

Reporting Summary

Nature Portfolio wishes to improve the reproducibility of the work that we publish. This form provides structure for consistency and transparency in reporting. For further information on Nature Portfolio policies, see our Editorial Policies and the Editorial Policy Checklist.

Statistics

For all statistical analyses, confirm that the following items are present in the figure legend, table legend, main text, or Methods section.

n/a	Confirmed	
☐	☒	The exact sample size (*n*) for each experimental group/condition, given as a discrete number and unit of measurement
☐	☒	A statement on whether measurements were taken from distinct samples or whether the same sample was measured repeatedly
☒	☐	The statistical test(s) used AND whether they are one- or two-sided<br>*Only common tests should be described solely by name; describe more complex techniques in the Methods section.*
☒	☐	A description of all covariates tested
☒	☐	A description of any assumptions or corrections, such as tests of normality and adjustment for multiple comparisons
☐	☒	A full description of the statistical parameters including central tendency (e.g. means) or other basic estimates (e.g. regression coefficient) AND variation (e.g. standard deviation) or associated estimates of uncertainty (e.g. confidence intervals)
☒	☐	For null hypothesis testing, the test statistic (e.g. *F*, *t*, *r*) with confidence intervals, effect sizes, degrees of freedom and *P* value noted<br>*Give P values as exact values whenever suitable.*
☒	☐	For Bayesian analysis, information on the choice of priors and Markov chain Monte Carlo settings
☒	☐	For hierarchical and complex designs, identification of the appropriate level for tests and full reporting of outcomes
☒	☐	Estimates of effect sizes (e.g. Cohen's *d*, Pearson's *r*), indicating how they were calculated

Our web collection on statistics for biologists contains articles on many of the points above.

Software and code

Policy information about availability of computer code

Data collection	No software was used for data collection
Data analysis	All analysis tools and scripts from raw sequence data are publicly available. Code for demultiplexing from raw sequence data is available here: https://github.com/adeylab/unidex, and code for all sciMETv2 processing from demultiplexed reads through all aspects of analysis are available here: https://github.com/adeylab/sciMETv2; additional code for plotting is available here: https://github.com/adeylab/scitools

For manuscripts utilizing custom algorithms or software that are central to the research but not yet described in published literature, software must be made available to editors and reviewers. We strongly encourage code deposition in a community repository (e.g. GitHub). See the Nature Portfolio guidelines for submitting code & software for further information.

Data

Policy information about availability of data

All manuscripts must include a data availability statement. This statement should provide the following information, where applicable:

- Accession codes, unique identifiers, or web links for publicly available datasets
- A description of any restrictions on data availability
- For clinical datasets or third party data, please ensure that the statement adheres to our policy

All raw sequence data has been deposited in the Neuroscience Multi-omic Data Archive (NeMO) the sciMETv2 collection within the BICCN RF1_Adey grant accession

under restricted use access (human genetic data for research use only) and can be obtained by requesting access through the NeMO portal. Methylation calls are also available from NeMO with open access under the same accession.

Human research participants

Policy information about studies involving human research participants and Sex and Gender in Research.

Reporting on sex and gender	Sex is provided for the assayed specimen.
Population characteristics	Specimen informaiton is provided. The specimen is form a healthy human female age 54.
Recruitment	Specimens were obtained from the Oregon Brain Bank.
Ethics oversight	The OHSU Brain Bank cohort is overseen by the OHSU Institutional Review Board and consented for genetic data sharing and genomic data under restricted access for research purposes only.

Note that full information on the approval of the study protocol must also be provided in the manuscript.

Field-specific reporting

Please select the one below that is the best fit for your research. If you are not sure, read the appropriate sections before making your selection.

☒ Life sciences ☐ Behavioural & social sciences ☐ Ecological, evolutionary & environmental sciences

For a reference copy of the document with all sections, see nature.com/documents/nr-reporting-summary-flat.pdf

Life sciences study design

All studies must disclose on these points even when the disclosure is negative.

Sample size	One human specimen was used for technology development purposes as well as mouse specimens for cell collision experiments. The focus of the study is technology development and therefore statistical power for biological conclusions was not a component.
Data exclusions	Data were from a single individual and no experimental replicates were excluded. Individual cells within the data were filtered to retain those with minimal coverage thresholds.
Replication	The study is technology development with multiple experiments demonstrating minimal experimental biases. Data were replicated on a separate specimen as well as integrated with publicly available data confirming no experimental bias.
Randomization	The study did not involve separate experimental groups as there were no biological hypotheses.
Blinding	The study did not involve blinding as there were no biological hypotheses and the work is centered on technology development and resulting data quality.

Reporting for specific materials, systems and methods

We require information from authors about some types of materials, experimental systems and methods used in many studies. Here, indicate whether each material, system or method listed is relevant to your study. If you are not sure if a list item applies to your research, read the appropriate section before selecting a response.

Materials & experimental systems

n/a	Involved in the study	
☒	☐	Antibodies
☒	☐	Eukaryotic cell lines
☒	☐	Palaeontology and archaeology
☐	☒	Animals and other organisms
☒	☐	Clinical data
☒	☐	Dual use research of concern

Methods

n/a	Involved in the study	
☒	☐	ChIP-seq
☒	☐	Flow cytometry
☒	☐	MRI-based neuroimaging

Animals and other research organisms

Policy information about studies involving animals; ARRIVE guidelines recommended for reporting animal research, and Sex and Gender in Research

Laboratory animals	Mouse C57BL6 brain tissue was used in the study as a cross-species control.
Wild animals	Wild animals were not used in the study.
Reporting on sex	Sex was not determined on the mouse brain sample used in this study. The tissue was used as a cross-species control, therefore sex is not relevant for the experimental aim.
Field-collected samples	Field collected samples were not used.
Ethics oversight	Samples were obtained as waste from unrelated experiments that were overseen by the OHSU IACUC

Note that full information on the approval of the study protocol must also be provided in the manuscript.
